# Diagnostic Accuracy and Complication of Computed Tomography (CT)-Guided Percutaneous Transthoracic Lung Biopsy in Patients 80 Years and Older

**DOI:** 10.3390/jcm11195894

**Published:** 2022-10-06

**Authors:** Yoon Joo Shin, Jeong Geun Yi, Donghee Son, Su Yeon Ahn

**Affiliations:** 1 Department of Radiology, Konkuk University Medical Center, Seoul 05030, Korea; 2 Department of Radiology, Konkuk University Medical Center, Konkuk University School of Medicine, Seoul 05030, Korea; 3 Research Coordinating Center, Konkuk University Medical Center, Seoul 05030, Korea

**Keywords:** image-guided biopsy, cancer of lung, elderly

## Abstract

This research evaluated the diagnostic accuracy and complication rate of computed tomography (CT)-guided percutaneous transthoracic lung biopsy (PTNB) in patients 80 years and older. The study sought to identify risk factors for diagnostic failures or complications of PTNBs. We examined 247 CT-guided PTNBs performed from January 2017 through December 2020, noting patient demographics, lesion or procedure types, pathology reports, and other procedure-related complications. Study groups were divided into two: one with patients aged 80 years and older (Group 1) and the other with patients aged 60 to 80 years (Group 2). The research first determined each groups’ diagnostic accuracy, sensitivity, specificity, diagnostic failure rate, and complication rate and then evaluated the risk factors for diagnostic failures and complications. The diagnostic accuracy, sensitivity, specificity, and diagnostic failure rates were 95.6%, 94.9%, 100%, and 18.9%, respectively, in Group 1. The overall and major complication rates in Group 1 were 29.6% and 3.7%, respectively. Lesion size was the only risk factor for diagnostic failure (adjusted odds ratio [OR], 0.46; 95% confidence interval [CI], 0.24–0.90). There was no significant risk factor for complications in Group 1. CT-guided PTNBs in patients 80 years and older indicate comparable diagnostic accuracy and complication rates.

## 1. Introduction

Lung cancer is a disease that affects mainly elderly patients, and the number of cancer diagnoses is expected to increase with the aging global population [1,2]. Statistics indicate that by 2050, the global elderly population will increase more than two-fold. Compared to 2020, the number of people aged over 60 is projected to double, reaching 2.1 billion, and the number of people aged 80 and over is projected to nearly triple, marking 426 million [3]. The modern medical environment allows more frequent chest CTs, increasing the chance of detecting malignant lung nodules in patients generally, but especially in elderly patients [4]. In the coming years, clinicians will likely encounter more lung cancer patients in the 80 years and older age-group, but this age bracket is underrepresented in existing clinical trials. Strategies must be implemented to prepare for evidence-based management of elderly patients with lung cancer [5,6,7].

A lung cancer patient 80 years or older generally has a poorer prognosis than patients of a younger age [8]. However, despite the poor prognosis, we cannot justify denial of surgeries or chemotherapy treatments for elderly patients [6]. Patients 80 years and older are underrepresented in most clinical studies and therefore do not offer clinicians sufficient guidance for conducting evidence-based case management. Moreover, in practice, more than half of patients 80 years and older choose not to undergo proper evaluation or refuse treatment for lung cancer because they are concerned about the side effects or are afraid of their individual performance status or cancer stage [9,10]. Therefore, it is in the patient’s interest, as well as in the interest of establishing the foundations for an effective case management system, that clinicians consider working closely with elderly patients. Clinicians should work with appropriately selected patients and emphasize the importance of participating in medical examinations and taking appropriate personalized medicine to improve their prognosis.

Percutaneous transthoracic needle lung biopsy (PTNB) is a well-established, minimally invasive procedure for evaluating lung abnormalities with excellent diagnostic accuracy and a low incidence of major complications [11,12]. According to previous studies, age can be a risk factor for complications, and other potential factors include emphysema, large needle, small lesions, increased lesion depth, and increased pleural passage [11,13,14]. However, few studies on the accuracy and safety of PTNB in patients 80 years and older [15,16] have been published, and moreover they fail to cover the diagnostic failure rates and their risk factors.

This study seeks to add to the existing literature by evaluating the diagnostic accuracy and complication rates of CT-guided PTNBs and identifying the risk factors associated with diagnostic failures and complications of PTNBs in patients 80 years and older.

## 2. Materials and Methods

This retrospective study was approved by the institutional review board of Konkuk University Medical Center. The requirement for written informed consent was waived because of the retrospective nature of the study.

### 2.1. Study Population

This study examined 247 biopsies of 227 consecutive patients aged 60 years and older who underwent CT-guided PTNB between January 2017 and December 2020. There were 35 patients who underwent multiple biopsies: six underwent repeat biopsies for the same target lesion after technical failure or nondiagnostic PTNB, and 29 underwent re-biopsies because of tumor progression requiring treatment regimen change.

### 2.2. Medical Record Review

Two thoracic radiologists (Y.J.S. and S.Y.A., with 4 and 7 years of experience in thoracic imaging, respectively) collected data from the electronic medical record system.

Patient-related demographics, including age, sex, smoking history (never-, former, or current smoker), and smoking status (pack-years) were documented. Associated CT findings, such as the presence or absence of emphysema and interstitial lung abnormality (ILA) or interstitial lung disease (ILD), were also investigated.

Lesion-related data included the size, the type of the lesion (solid, part-solid, ground-glass opacity (GGO)), and the location of the lesion (lower lobe vs. upper/middle lobe). The maximum diameter of the target lesion was measured on the axial preprocedural chest CT image.

Procedure-related data included the type of biopsy procedure (fine needle aspiration (FNA), core needle biopsy (CNB), or both), biopsy needle size (18, 20, or 22 G), depth of needle pathway, number of tissue samplings, presence of transfissural approach, location of the needle tip within the target (yes or no), and position of the patient (supine, prone, others) during the procedural CT scan. The depth of the needle pathway was measured as the distance from the pleura to the target lesion along the needle pathway on the procedural CT image.

The pathological reports of the PTNBs were categorized as positive, negative, and nonevaluable results, the last being nonevaluable due to insufficient specimens for diagnostic accuracy calculation. Positive results were “malignancy,” “specific benign,” “atypical cells suggestive of malignancy,” “atypical cells suspicious for malignancy,” and “atypical cells of indeterminate malignancy.” Negative results were “aspecific benign” and “atypical cells favoring benign.” Nonevaluable results included pathological reports that indicated that the specimen was inadequate or insufficient for pathological diagnosis.

During the analysis, technical failure, diagnostic errors (false positive or false negative), and nondiagnostic specimens were considered “diagnostic failure.” True-positive and true-negative results were considered “diagnostic success” [17,18].

PTNB-related complications were recorded under two categories (minor and major), according to the Society of Interventional Radiology Guidelines [11,19], with minor modifications. Minor complications included pneumothorax without requiring drainage and transient hemoptysis. A minor pulmonary hemorrhage without symptoms was not regarded as a complication. Major complications included pneumothorax requiring interventions such as percutaneous drainage tube insertion, massive hemoptysis requiring embolization, hemothorax, acute exacerbation of chronic obstructive pulmonary disease (COPD) or ILD, air embolism, and death. This study included technical failure due to pneumothorax in the complication analysis, and it was assumed that acute exacerbation within 1 month of the biopsy would be related to the PTNB procedure.

In addition, the research evaluated the length of the hospitalization period during the patient’s admittance for the procedure and deaths of patients within 90 days after PTNB. The number of hospitalization days was calculated by subtracting the date of biopsy from the date of discharge. In cases of death, the date of survival was calculated by subtracting the date of biopsy from the date of death.

### 2.3. Final Diagnosis

The final diagnosis was made by two thoracic radiologists (Y.J.S. and S.Y.A) based on a complete review of serial chest CTs and medical records using one of the following methods [20,21]: (a) pathological results were used for diagnosis when the lesion was surgically resected, (b) nonsurgical biopsy results were accepted when the results indicated malignancy or specific benign pathological findings, (c) lesions were considered malignant if they showed obvious malignant clinical behavior during the follow-up period (e.g., increased lesion size, disease progression such as developing metastasis, lesion regression after chemotherapy), and (d) lesions were considered benign if the size decreased by 20% or more in diameter or was stable for at least 2 years. Lesions not diagnosed were excluded from the diagnostic accuracy analysis, but were included in the complication rate analysis.

### 2.4. Biopsy Procedure

Board-certificated thoracic radiologists with 4–30 years of experience performed all procedures. All biopsy procedures were performed as a component of routine clinical practice using a conventional CT scanner (GE LightSpeed VCT, GE Healthcare, USA [from 2008 to 2019] and Siemens Somatom Definition, Siemens Medical Solutions, Malvern, PA, USA [from 2019 to 2020]). Before the procedure, the patient was instructed regarding breath holding during inspiration or expiration if needed. The patient underwent a biopsy procedure under local anesthesia without sedation. Biopsy needles were inserted in a “stop and go” manner that discontinuously checks the needle path: inserting a needle, checking needle location, and advancing or changing the needle path. Specimens were obtained via FNA (22-gauge Chiba biopsy needle; Cook Medical, Indiana, USA) or CNB (18- or 20-gauge cutting needle; Starcut; TSK Laboratory, Tochigi, Japan). A coaxial technique was exclusively used for CNB using the Starcut needle. The type and size of biopsy needles were selected according to the characteristics of the target lesion and the preference of the radiologist performing the procedure. Immediate on-site pathological evaluation of tissue samples was unavailable. Immediate postprocedural CT images were acquired to check for procedure-related complications, followed by chest radiographs within 1 h of the procedure and another follow-up radiograph within 1 day postprocedure. Patients were instructed to lie still during recovery. In our institution, all patients were hospitalized the day before or on the day of PTNB. In general, if there were no complications, patients were discharged the day of or 1 day after the procedure.

### 2.5. Statistical Analysis

Patients were classified into two groups, one group of patients aged 80 years and older (Group 1) and another group of patients aged between 60 and 80 years (Group 2). Continuous values are presented as means ± standard deviation (SD) and numbers (%) and tested using an independent *t*-test. Categorical values are presented as numbers (%) and tested using the chi-squared test or Fisher’s exact test.

All analyses were performed on a per-biopsy basis. Biopsies with technical failure or undecided final diagnoses were excluded when calculating the diagnostic accuracy. Biopsies with technical failure were included when calculating the complication rate.

The diagnostic accuracy, sensitivity, and specificity of CT-guided PTNBs were calculated. “Nonevaluable results due to insufficient specimens” were considered false negative when calculating sensitivity and false positive when calculating specificity, according to the intention-to-diagnose principle [22].

The complication rates of PTNB were categorized as overall, major, and minor. Values are presented as numbers (%) and were tested by chi-squared test or Fisher’s exact test. Univariate and multivariate logistic regression analyses analyzed risk factors for diagnostic failure and for complications. Variables with a *p*-value < 0.1 in the univariate analyses were included in the multivariate analysis. A *p*-value <0.05 was considered statistically significant in the multivariate analysis. Statistical analyses were performed using the commercially available R software version 4.2.0.

## 3. Results

### 3.1. Patient Demographics

The patient demographics are shown in Table 1. Among the total of 247 PTNB biopsies, there were 54 biopsies of patients aged 80 years and older (Group 1) and 193 biopsies of patients younger than 80 years (Group 2). Group 1 had a higher number of female patients, presence of ILA or ILD, and presence of biopsy needle traversing emphysema or lung fibrosis than Group 2 (*p* = 0.017, 0.048, and 0.029, respectively). There was no significant difference in nodule type, size, location, or other variables between the two groups.

### 3.2. Diagnostic Accuracy

Of the total 247 PTNBs, 13 PTNBs had technical failures, five undetermined final diagnosis due to short follow-up, and the pathological report was unavailable in one. Those PTNBs were excluded from the diagnostic accuracy analysis. The pathological reports for the PTNBs indicated that of the remaining 228 procedures, 181 (37/45, and 144/183 in Group 1 and Group 2, respectively) had malignant results, 39 (8/45 and 31/183, respectively) had benign results, and 8 (0/45, and 8/183, respectively) had nonevaluable results due to insufficient specimens. The final diagnoses were established as malignant and benign lesions in 39 (86.7%) and six (13.3%) in Group 1 and 160 (87.4%) and 23 (12.6%) in Group 2, respectively. Therefore, the diagnostic accuracy, sensitivity, and specificity were 95.6% (43/45), 94.9% (37/39), and 100% (6/6) in Group 1, 92.6% (162/175), 87.7% (143/163), and 67.9% (19/27) in Group 2, and 93.2% (205/220), 89.1% (180/202), and 73.5% (25/34) in the total population. We present detailed results after the subgroup analysis of CNB and FNA patients in Table 2. Since Group 1 had a small number of FNA patients, statistical comparison was not feasible.

Of 228 patients with a determined final diagnosis, lung cancer was the most common final diagnosis. A total of 184 patients were diagnosed with lung cancer: 82.2% of Group 1 and 80.3% of Group 2. Table 3 outlines the detailed categories of final diagnoses.

### 3.3. Diagnostic Yield

The diagnostic success and failure rates were calculated after excluding patients with uncertain final diagnoses (*n* = 5) and patients with unavailable final diagnoses (*n* = 1). The diagnostic failure rate was 18.9% (10/53) in Group 1, comprising eight cases of technical failure and two cases of false-negative results, and 13.8% (26/188) in Group 2, comprising five cases of technical failure, one case of false-positive results, 12 cases of false-negative results, and eight cases of insufficient specimens. There was no significant difference in diagnostic failure rates between the two groups, but the technical failure rate was significantly higher in Group 1 than in Group 2. In 13 biopsies (5.3%), the coaxial introducer tip or needle failed to reach the target lesion and obtain specimen samples. Eight failed biopsies were found in Group 1 (14.8%) and five in Group 2 (2.6%). In Group 1, biopsy failed in four patients due to pneumothorax, and four patients failed to cooperate with the biopsy procedure due to dementia, poor hearing capability, or poor general condition. In Group 2, three patients developed pneumothorax, and two failed due to uncontrolled breath holding. After multivariate analysis, not only lesion size (adjusted odds ratio [AOR], 0.36; 95% confidence interval [CI], 0.19–0.67) but also older age (8.12; 2.33–28.23) were risk factors for technical failure.

### 3.4. Complications

The overall complication rate was 36.4% (90/247) in total, with 29.6% (16/54) in Group 1 and 38.3% (74/192) in Group 2 patients. In Group 1, minor and major complication rates were 25.9% (14/54) and 3.7% (2/54), respectively, and in Group 2, the rates were 35.2% (68/193) and 3.1% (6/193), respectively (Table 4). Major complications, including pneumothorax requiring chest tube drainage, occurred in 3.7% and 2.6% of patients in Groups 1 and 2, respectively, and hemothorax occurred in 0 and 0.5% of patients in Groups 1 and 2, respectively. Pneumothorax that did not require chest tube drainage occurred in 25.9% of patients in Group 1 and 33.7% of patients in Group 2. Hemoptysis and hemothorax were observed only in Group 2. There were no other major complications, including air embolism, death, or acute exacerbation of COPD or ILD in both groups. All patients showed pneumothorax immediately after or within 1 h following the PTNB on postprocedure chest radiograph, and there were no patients with delayed pneumothorax. No statistically significant differences between the two groups were observed for any complications.

### 3.5. Risk Factors for Diagnostic Failure and Complications

In multivariate analysis, only lesion size (0.71; 0.55–0.90) increased the risk of diagnostic failure in the total patient population. Age of 80 years or older did not increase the risk of diagnostic failure (*p* = 0.358). In addition, lesion size (0.46; 0.24–0.90) was associated with diagnostic failure in Group 1 patients (Table 5).

The transfissural approach (5.69; 1.67–19.5) and lesion size (0.83; 0.70–0.99) were independent risk factors for overall complications in total patients. Female sex (1.90; 1.00–3.60) and needle traversing lung fibrosis or emphysema (3.69; 1.20–11.3) were risk factors for pneumothorax occurrence in the total patient population. However, when subgroup analysis was performed in Group 1, there was no statistically significant risk factor for overall complications, minor or major complications, or pneumothorax occurrence (Table 6).

### 3.6. Hospitalization Days and Death

The mean number of hospitalization days was 9.4 ± 14.7 days in Group 1 and 6.0 ± 10.4 days in Group 2 (*p* = 0.116). As routine practice, patients were discharged from the hospital the day of or the day after the PTNB procedure. However, the hospitalization period was extended due to the occurrence of complications after the procedure, additional treatment, including surgery or chemotherapy, and management of comorbid diseases such as cardiac disease and diabetes mellitus.

**Figure 1:**
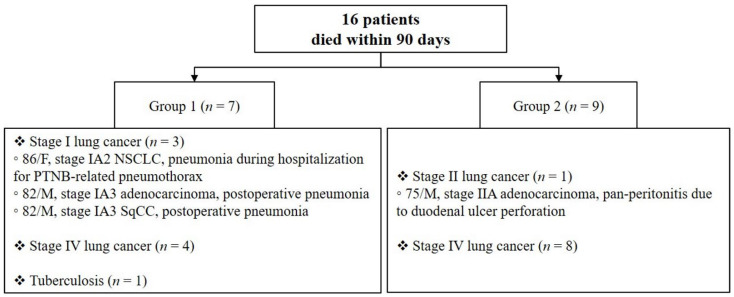
Detailed information of patients who died within 90 days after CT-guided PTNB. Patient 1 was an 86-year-old female with stage IA2 non-small-cell lung cancer who developed pneumonia while hospitalized for pneumothorax care after PTNB. She expired 49 days after PTNB and did not receive any treatment for lung cancer. She was a nonsmoker, but had emphysema, idiopathic pulmonary fibrosis (IPF), diabetes mellitus (DM), chronic hepatitis C, and chronic kidney disease. Patient 2 was an 82-year-old male with stage IA3 adenocarcinoma. He underwent lobectomy and developed pneumonia during postoperative management. He died 52 days after PTNB and 32 days after the surgery. A 50 pack-year former smoker, patient 2 also had emphysema, early IPF, and hypertension. Patient 3 was an 82-year-old male with stage IA3 squamous cell carcinoma. He received a wedge resection and was rehospitalized for pneumonia the day following his discharge from surgery. He died 77 days after PTNB and 63 days after the surgery. He was a 200 pack-year former smoker with emphysema, early IPF, coronary artery disease, DM, hypertension, Alzheimer’s disease, and liver cirrhosis. Patient 4 was a 75-year-old male with stage IIA adenocarcinoma and underwent lobectomy immediately after PTNB, along with adjuvant chemotherapy. He was admitted to the emergency room for duodenal ulcer perforation and pan-peritonitis and died of multiorgan failure 83 days after PTNB. He was a 40 pack-year former smoker and had emphysema, a history of ST-elevation myocardial infarction, hypertension, DM, and chronic kidney disease.

Sixteen patients died within 90 days after PTNB, as shown in Figure 1. Of them, seven were in Group 1, including three with stage I, three with stage IV lung cancer, and one with tuberculosis. Pneumonia was the cause of death in three patients with stage I lung cancer, and all three patients had comorbid diseases, including emphysema and idiopathic pulmonary fibrosis (IPF). Nine patients were in Group 2, including one with stage II, five with stage IVA, and three with stage IVB lung cancer. Pan-peritonitis and multiorgan failure were the causes of death in patients with stage II lung cancer.

## 4. Discussion

This retrospective study aimed to evaluate the diagnostic accuracy and complication rate of CT-guided PTNB in patients 80 years and older (Group 1), with a concurrent comparison group consisting of patients aged 60 to 79 years (Group 2). Diagnostic accuracy, sensitivity, and specificity showed slightly higher values in Group 1 than in Group 2 (95.6%, 94.9%, 100 vs. 92.6%, 87.7%, 67.9%, respectively) without statistically significant difference. The diagnostic failure rate was not different between the two groups (18.9% vs. 13.8%), but the technical failure rate was significantly higher in Group 1 than in Group 2 (15.1% vs. 2.7%). The overall complication rate and major complication rates in Group 1 and Group 2 were 29.6% vs. 38% and 3.7% vs. 3.1%, respectively, without a statistically significant difference between the two groups. In elderly patients 80 years and older, small lesion was the only risk factor for diagnostic failure, and there was no significant risk factor for complications.

Overall diagnostic accuracy, sensitivity, and specificity in this study population were 93.2%, 89.1%, and 73.5%, respectively. CT-guided PTNB is an accurate diagnostic procedure for pulmonary nodule evaluation, with known diagnostic accuracy of 90.0–97.0%, sensitivity of 92.5–95.7%, and specificity of 86.5–100% [12,21,23]. When nondiagnostic results were considered to evaluate diagnostic performance, the sensitivity and specificity decreased by 4.5% and 10.7%, respectively [24]. In this regard, the accuracy of CT-guided PTNBs in patients 80 years and older was reasonable. Nonevaluable results due to insufficient specimens applied to eight cases, only in Group 2, which might have caused the lower diagnostic performance in Group 2. Moreover, medical decision-making processes often tend to prefer patients with suspected malignancy or with a target lesion that is easily accessible and less risky for biopsy procedure selection, especially in patients aged 80 years and older. Therefore, care should be taken in interpreting results regarding elderly patients, especially those aged 80 years and older.

The diagnostic failure rate, specifically technical failure rate, was not negligible in this study. Diagnostic failures of PTNBs were divided into technical failure, false-positive, false-negative, and nonevaluable results due to insufficient specimens. For eight patients of Group 1 and five patients of Group 2, target tissues due to persistent pneumothorax or poor cooperation of the patient had not been obtained. Breath holding and positioning are mandatory for patients during the PTNB procedure, but in this study, some of the patients failed to hold their breath or maintain a prone or decubitus position, due to hearing difficulty, dementia, or poor general condition. The diagnostic failure rate in this study was slightly higher than that in previous studies [12,17,18]. There were several differences between previous and current studies. First, this study’s average age was higher since it only included patients aged over 60 years. Second, technical failure or technical error was not included in the large-scale multicenter study [12]. Third, the guiding modalities were various, including CT, cone-beam CT, and fluoroscopy in other studies. In addition, the rate of emphysema, which can be a risk factor for diagnostic failure, was more than twice as high in this study group (39.7%) than in other studies.

Small lesion was the only risk factor for diagnostic failure in the total population and Group 1 patients. This finding is consistent with previous studies that suggested that smaller lesions were associated with an increased risk of diagnostic failure [12,25]. Several factors have been reported as risk factors for diagnostic failure in previous studies (i.e., use of FNA only [12], subsolid lesions [12], lower lobe location [25], or occurrence of pneumothorax during the procedure [25]) were not found to be statistically associated with diagnostic failure in the current study.

Complications in this study are comparable with previous studies reporting that pneumothorax rates were 7.5–38.8% and rates of pneumothorax requiring intervention were 1.8–5.6% [11,13,14,15,16,26,27,28]. A recent meta-analysis regarding the complication rate of CT-guided PTNBs showed overall complication rates of 38.8% and 24.0% in core biopsy and FNA, with major complication rates of 5.7% and 4.4%, respectively [11]. The pneumothorax rate and pneumothorax requiring intervention rates were 25.3% and 5.6% in core biopsy and 18.8% and 4.3% in FNA, respectively. In Group 1, only pneumothorax requiring intervention occurred as a major complication, occurring in two patients (3.7%) and resulting in technical failure.

It is worth noting is that there was no significant difference in pneumothorax or complication rates between the two groups, whereas previous studies showed a strong correlation between older age and pneumothorax or complication rates [13,14,26,29]. However, this difference may be due to different cutoff values for age, different comparison age-groups, and different guiding modalities of PTNB. The current study showed results consistent with a previous study that reported a higher risk of complications in patients in their 60s and 70s and lower in patients in their 80s or older [15,27]. In addition, Tongbai et al. showed that the pneumothorax rate was not significantly different between over-80 and under-80 patient groups (23% vs. 24%) [16]. Although the study population of Group 1 was small and the analysis was limited, the lower complication rate and pneumothorax rate in Group 1 may be because the patients in this study were more carefully selected for the biopsy. Selection bias can arise in old age, especially in elderly patients. Since elderly patients are expected to show poor overall performance, worse prognosis, and worse complications, in patients with a high possibility of biopsy failure or complications, a biopsy may not be attempted.

There was no statistically significant risk factor for overall, minor complications, or major complications in Group 1 patients. A transfissural approach and small lesion were risk factors for overall complications in all patients, and female sex and needle traversing lung fibrosis or emphysema were risk factors for pneumothorax occurrence, which is partially consistent with previous studies. Previous studies showed that older age, presence of emphysema, length from the pleura, multiple pleural passages, and small lesion were significant risk factors for pneumothorax occurrence [11,25,28,29].

In addition to complications, we assessed the duration of hospital stay to determine whether there was an extension of hospital stay, and we examined whether death occurred within 90 days after PTNB to investigate unexpected death during the follow-up period. The mean hospitalization periods were slightly longer in Group 1 than in Group 2, but suggested no clinical significance. Unexpected deaths were investigated, including those diagnosed with early lung cancer or benign disease but died early (within 90 days after being diagnosed with PTNB). As a result, 16 patients, including four early lung cancer patients, died within 90 days, suggesting that early diagnosis of lung cancer may not always be beneficial to elderly patients with many comorbidities. Among the four early lung cancer patients who died within 90 days, three Group 1 patients died while hospitalized for lung cancer-related procedures or surgery, and one Group 2 patient died due to peritonitis unrelated to lung cancer.

Limitations of this study and its method do exist. First, the retrospective nature of the study suggests some limitations. Second, selection bias may exist in both approaches, with clinicians not referring to patients with a high risk of diagnostic failure or complications due to biopsy, and also elderly patients not willing to undergo further examinations themselves. Third, this study did not evaluate performance status or comorbid disease, which could significantly affect the prognosis of elderly patients. However, we did evaluate relevant CT findings for COPD and ILD and investigated comorbidities in the cases of patients who died within 90 days. Objective measurements, such as Comprehensive Geriatric Assessment (CGA) or Eastern Cooperative Oncology Group (ECOG) performance status, may help ascertain the patient’s exact clinical status when determining diagnosis or treatment process [30]. There was also potential operator bias; however, there was no preference or tendency for radiologists to perform more or fewer biopsies on patients over 80 years of age. Five dedicated thoracic radiologists performed the biopsies, and for each radiologist, approximately 20–23% of the biopsies were performed on patients aged 80 years and older. Lastly, some risk factors for diagnostic failure or complications may have failed to show statistical significance due to the small sample.

## 5. Conclusions

In conclusion, this study suggests that CT-guided PTNB in patients 80 years and older shows comparable diagnostic accuracy and complication rates. Patients 80 years and older were potentially at much higher risk of adverse events, but were not at an increased risk of major complications. Proceeding with CT-guided PTNB requires extra caution before, during, and after the procedure because of the high technical failure rate and possible sudden changes in health conditions or prognosis. Sensible and well-advised CT-guided PTNB in patients 80 years and older may play a vital role in this era of demographic changes and an aging population.

## Figures and Tables

**Table 1:** Demographics of patients who underwent CT-guided PTNB.

Characteristics	Total (*n* = 247)	60 ≤ Age < 80 (*n* = 193)	Age ≥ 80 (*n* = 54)	*p* Value
Age	72.6 ± 7.81	69.48 ± 5.52	83.76 ± 3.22	<0.001
Sex				
Male	166 (67.2)	137 (71.0)	29 (53.7)	0.017
Female	81 (32.8)	56 (29.0)	25 (46.3)	
Smoking status				
Never-smoker	85 (37.1)	63 (35.2)	22 (44.0)	0.161
Former smoker	89 (38.9)	68 (38.0)	21 (42.0)	
Current smoker	55 (24.0)	48 (26.8)	7 (14.0)	
Smoking pack-years	39.8 ± 27.2	36.7 ± 20.7	52.0 ± 43.2	0.010
Emphysema				
No	149 (60.3)	120 (62.2)	29 (53.7)	0.261
Yes	98 (39.7)	73 (37.8)	25 (46.3)	
ILA or ILD				
No	205 (83.0)	165 (85.5)	40 (74.1)	0.048
Yes	42 (17.0)	28 (14.5)	14 (25.9)	
Location				
Upper lobe	124 (50.2)	92 (47.7)	32 (59.3)	0.132
Middle/lower lobes	123 (49.8)	101 (52.3)	22 (40.7)	
Target size (cm)	4.04 ± 2.11	4.04 ± 2.18	4.03 ± 1.87	0.972
Type				
Solid	236 (95.5) *	186 (96.4)	50 (92.6)	0.401
Part-solid	10 (4.0) *	6 (3.1)	4 (7.4)	
Pure GGN	1 (0.4) *	1 (0.5)	0 (0.0)	
Needle traversing emphysema or lung fibrosis				
No	221 (92.5)	179 (94.7)	42 (84.0)	0.029
Yes	18 (7.5)	10 (5.3)	8 (16.0)	
Needle tip in the target				
No	27 (11.3)	19 (10.1)	8 (16.0)	0.237
Yes	212 (88.7)	170 (89.9)	42 (84.0)	
Pleura to target(cm)	1.23 ± 1.43	1.24 ± 1.45	1.17 ± 1.37	0.770
Position				
Supine	75 (31.4) *	57 (30.2) *	18 (36.0)	0.140
Prone	144 (60.3) *	119 (63.0) *	25 (50.0)	
Others	20 (8.4) *	13 (6.9) *	7 (14.0)	
Biopsy needle				
FNA only	44 (18.4)	35 (18.5)	9 (18.0)	0.933
CNB or combined	195 (81.6)	154 (81.5)	41 (82.0)	
Size of needle				
22 G	46 (19.2)	35 (18.5)	11 (22.0)	0.835
20 G	112 (46.9)	90 (47.6)	22 (44.0)	
18 G	81 (33.9)	64 (33.9)	17 (34.0)	
Transfissural approach				
No	223 (93.3)	178 (94.2)	45 (90.0)	0.338
Yes	16 (6.7)	11 (5.8)	5 (10.0)	

* Unless otherwise specified, the data are number of biopsies (and the percentages). CNB, core needle biopsy; FNA, fine needle aspiration; ILA, interstitial lung abnormality; ILD, interstitial lung disease; IQR, interquartile range. * Because of rounding, percentages may not total 100.

**Table 2:** Subgroup analysis for CNB and FNA.

		Diagnostic Accuracy	Sensitivity	Specificity
CNB	Group 1	94.7 (36/38)	94.1 (32/34)	100 (4/4)
	Group 2	93.8 (136/145)	89.6 (121/135)	75 (15/20)
	Total	94.0 (172/183)	90.5 (153/169)	79.2 (19/24)
FNA	Group 1	100 (7/7)	100 (5/5)	100 (2/2)
	Group 2	86.7 (26/30)	78.9 (22/28)	50 (4/8)
	Total	89.2 (33/37)	81.8 (27/33)	60 (6/10)
Group 1		95.6 (43/45)	94.9 (37/39)	100 (6/6)
Group 2		92.6 (162/175)	87.7 (143/163)	67.9 (19/27)
Total		93.2 (205/220)	89.1 (180/202)	73.5 (25/34)

CNB, core needle biopsy; FNA, fine needle aspiration.

**Table 3:** Final Diagnosis of Study Population.

Diagnosis	Age ≥ 80 (*n* = 45)	Age < 80 (*n* = 183)
Lung cancer	37 (82.2%)	147 (80.3%)
Adenocarcinoma	25 (55.6%)	84 (45.9%)
Squamous cell carcinoma	8 (17.8%)	36 (19.7%)
NSCLC, NOS	4 (8.9%)	21 (11.5%)
Small cell carcinoma	0 (0%)	5 (2.7%)
Metastasis	1 (2.2%)	8 (4.4%)
Other malignancy	1 (2.2%)	6 (3.3%)
Benign	6 (13.3%)	23 (12.6%)
Tuberculosis	3 (6.7%)	10 (5.5%)
Pneumonia or others	3 (6.7%)	13 (7.1%)

NSCLC, non-small-cell lung cancer; NOS, not otherwise specified.

**Table 4:** Complications of CT-guided PTNB in the study population.

Variable	Total (*n* = 247)	60 ≤ Age < 80 (*n* = 193)	Age ≥ 80 (*n* = 54)	*p* Value
Overall complications	90 (36.4)	74 (38.3)	16 (29.6)	0.240
Minor complications	82 (33.2)	68 (35.2)	14 (25.9)	0.199
Pneumothorax	79 (32.0)	65 (33.7)	14 (25.9)	0.280
Hemoptysis	7 (2.8)	7 (3.6)	0 (0.0)	0.352
Major complications	8 (3.2)	6 (3.1)	2 (3.7)	0.688
PCD due to pneumothorax	7 (2.8)	5 (2.6)	2 (3.7)	0.649
Hemothorax	1 (0.4)	1 (0.5)	0 (0.0)	1.000

PCD, percutaneous catheter drainage.

**Table 5:** Risk factors of diagnostic failure of CT-guided PTNB in total patients and Group 1 patients.

	Total Patients				Group 1			
Variable	Univariate		Multivariate		Univariate		Multivariate	
	Unadjusted OR	*p*-Value *	Adjusted OR (95% CI)	*p*-Value ^†^	Unadjusted OR	*p*-Value *	Adjusted OR (95% CI)	*p*-Value ^†^
	(95% CI)				(95% CI)			
Age								
60 ≤ Age < 80	Ref.		Ref.					
Age ≥ 80	1.449 (0.649–3.235)	0.365	1.470 (0.646–3.345)	0.358				
Sex								
Male	Ref.				Ref.			
Female	1.686 (0.815–3.489)	0.159			2.083 (0.512–8.472)	0.305		
Smoking status								
Never	Ref.				Ref.			
Former	1.077 (0.582–1.991)	0.813			2.235 (0.363–13.782)	0.386		
Current	1.076 (0.534–2.168)	0.838			1.583 (0.121–20.684)	0.726		
Smoking pack-years	0.998 (0.978–1.018)	0.808			0.993 (0.955–1.032)	0.713		
Smoking group								
≥1 PY	Ref.				Ref.			
≥30 PY	1.020 (0.334–3.119)	0.972			N/A	-		
Emphysema								
No	Ref.				Ref.			
Yes	1.783 (0.874–3.634)	0.112			1.150 (0.290–4.557)	0.842		
ILA or ILD								
No	Ref.				Ref.			
Yes	0.732 (0.267–2.010)	0.545			1.247 (0.274–5.678)	0.776		
Location								
Upper	Ref.				Ref.		Ref.	
Middle/lower lobes	1.750 (0.848–3.609)	0.13			4.833 (1.084–21.558)	0.039	2.660 (0.513–13.785)	0.244
Target size	0.707 (0.552–0.904)	0.006	0.706 (0.552–0.903)	0.006	0.417 (0.217–0.802)	0.009	0.463 (0.239–0.898)	0.023
Type								
Solid	Ref.				Ref.			
Part-solid	0.700 (0.085–5.772)	0.74			N/A	-		
Pure GGN	N/A	-						

GGN, ground-glass nodule; ILA, interstitial lung abnormality; ILD, interstitial lung disease; N/A, not applicable; OR, odds ratio; PY, pack-year. * *p* values from univariate analyses. † *p* values from multivariate analysis.

**Table 6:** Risk factors of overall complications of CT-guided PTNB in total patients and Group 1 patients.

	Total Patients				Group 1			
Variable	Univariate		Multivariate		Univariate		Multivariate	
	Unadjusted OR	*p*-Value *	Adjusted OR (95% CI)	*p*-Value ^†^	Unadjusted OR	*p*-Value *	Adjusted OR (95% CI)	*p*-Value ^†^
	(95% CI)				(95% CI)			
Age								
60 ≤ Age < 80	Ref.		Ref.		N/A			
Age ≥ 80	0.677 (0.353–1.300)	0.241	0.628 (0.297–1.328)	0.223	N/A			
Sex								
Male	Ref.				Ref.			
Female	1.421 (0.823–2.453)	0.207			1.768 (0.544–5.748)	0.344		
Smoking Status								
Never	Ref.				Ref.			
Former	1.077 (0.582–1.991)	0.813			2.000 (0.558–7.162)	0.287		
Current	1.076 (0.534–2.168)	0.838			N/A	-		
Smoking group								
0 PY	Ref.							
0 < PY <30	0.774 (0.344–1.742)	0.536			Ref.			
≥ 30 PY	1.172 (0.638–2.151)	0.609			1.600 (0.147–17.411)	0.7		
Emphysema								
No	Ref.				Ref.			
Yes	1.270 (0.750–2.152)	0.374			1.235 (0.383–3.981)	0.723		
ILA or ILD								
No	Ref.				Ref.			
Yes	1.230 (0.623–2.425)	0.551			2.250 (0.627–8.074)	0.214		
Location								
Upper	Ref.				Ref.			
Middle/lower lobes	1.544 (0.916–2.602)	0.103			1.193 (0.365–3.892)	0.77		
Target size	0.765 (0.655–0.894)	0.001	0.834 (0.703–0.989)	0.037	0.763 (0.534–1.091)	0.138		
Type								
Solid	Ref.				Ref.			
Part-solid	0.748 (0.188–2.966)	0.679			0.778 (0.075–8.095)	0.833		
Pure GGN	N/A	-						
Needle traversing								
lung fibrosis								
No	Ref.		Ref.		Ref.			
Yes	2.939 (1.095–7.887)	0.032	2.287 (0.765–6.840)	0.139	1.338 (0.277–6.458)	0.717		
Needle tip in the target								
No	Ref.				Ref.		Ref.	
Yes	0.699 (0.311–1.569)	0.385			0.213 (0.044–1.042)	0.056	0.508 (0.060–4.334)	0.536
Pleura to target (cm)	1.348 (1.118–1.625)	0.002	1.177 (0.958–1.445)	0.121	1.263 (0.820–1.943)	0.289		
Patient position								
Supine	Ref.				Ref.		Ref.	
Prone	1.313 (0.728–2.370)	0.366			3.929 (0.903–17.082)	0.068	2.611 (0.516–13.203)	0.246
Others	1.739 (0.636–4.753)	0.281			2.000 (0.256–15.623)	0.509	1.554 (0.182–13.239)	0.687
Needle type								
FNA only	Ref.				Ref.			
CNB or combined	0.909 (0.464–1.783)	0.782			0.929 (0.200–4.306)	0.925		
Size of needle								
22 G	Ref.				Ref.			
20 G	0.948 (0.465–1.933)	0.883			1.244 (0.251–6.174)	0.789		
18 G	1.058 (0.501–2.234)	0.883			1.455 (0.277–7.637)	0.658		
Transfissural approach								
No	Ref.		Ref.		Ref.		Ref.	
Yes	5.803 (1.810–18.602)	0.003	5.693 (1.665–19.468)	0.006 *	11.000 (1.115–108.488)	0.04	N/A	-
Number of tissue samplings								
≤2	Ref.				Ref.			
≥3	0.706 (0.263–1.896)	0.49			0.939 (0.088–10.003)	0.959		

CNB, core needle biopsy; DLP, dose length product; FNA, fine needle aspiration; *GGN*, ground-glass nodule; ILA, interstitial lung abnormality; ILD, interstitial lung disease; N/A, not applicable; OR, odds ratio; PY, pack-year. * *p* values from univariate analyses. ^†^ *p* values from multivariate analysis.

## Data Availability

The datasets generated during and/or analyzed during the current study are available from the corresponding author on reasonable request.
